# Impact of alkaloids in food consumption, metabolism and survival in a blood-sucking insect

**DOI:** 10.1038/s41598-020-65932-y

**Published:** 2020-06-10

**Authors:** Ignacio J. Muñoz, Pablo E. Schilman, Romina B. Barrozo

**Affiliations:** 1 Grupo de Neuroetología de Insectos Vectores, Laboratorio Fisiología de Insectos, Instituto Biodiversidad Biología Experimental Aplicada, CONICET; Departamento Biodiversidad Biología Experimental, Facultad Ciencias Exactas y Naturales, Universidad de Buenos Aires, UBA, Buenos Aires, Argentina; 2 Laboratorio de Ecofisiología de Insectos, Instituto de Biodiversidad y Biología Experimental y Aplicada, CONICET; Departamento de Biodiversidad y Biología Experimental, Facultad de Ciencias Exactas y Naturales, Universidad de Buenos Aires, UBA, Buenos Aires, Argentina

**Keywords:** Neuroscience, Feeding behaviour, Gustatory system

## Abstract

The sense of taste provides information about the “good” or “bad” quality of a food source, which may be potentially nutritious or toxic. Most alkaloids taste bitter to humans, and because bitter taste is synonymous of noxious food, they are generally rejected. This response may be due to an innate low palatability or due to a malaise that occurs after food ingestion, which could even lead to death. We investigated in the kissing bug *Rhodnius prolixus*, whether alkaloids such as quinine, caffeine and theophylline, are merely distasteful, or if anti-appetitive responses are caused by a post-ingestion physiological effect, or both of these options. Although anti-appetitive responses were observed for the three alkaloids, only caffeine and theophylline affect metabolic and respiratory parameters that reflected an underlying physiological stress following their ingestion. Furthermore, caffeine caused the highest mortality. In contrast, quinine appears to be a merely unpalatable compound. The sense of taste helps insects to avoid making wrong feeding decisions, such as the intake of bitter/toxic foods, and thus avoid potentially harmful effects on health, a mechanism preserved in obligate hematophagous insects.

## Introduction

All animals favor the intake of rich energy food and avoid the consumption of toxic compounds. Thereby, the sense of taste allows animals, including man, to evaluate and to predict the quality of a food source, which may be potentially nutritious or harmful. Thus, this sensory system plays a preponderant role in feeding because it triggers appetitive or aversive behaviors.

Many plants avoid insect herbivory by producing secondary metabolites that repel insects or have toxic effects^1^.Some noxious compounds are both unpalatable and toxic for insects whereas others are merely unpalatable or toxic^2^. To humans, many of these compounds taste bitter and are therefore known as bitter compounds. Particularly, insects have an aversive response to many alkaloids^3–6^. These compounds can reduce the amount of ingested food before being consumed (pre-ingestive mechanisms) or after being consumed (post-ingestive mechanisms)^2,7^. Feeding on harmful food can be prevented primarily with the aid of the taste system. Most insects studied so far have gustatory receptor neurons (GRNs) that are activated by noxious compounds and mediate deterrent/aversive responses^5,8–13^ etc. The responses of bitter-sensitive neurons depend on combinations and interactions between the taste receptors expressed on these neurons^14,15^. Besides, other gustatory neurons tuned to appetitive tastants, may modify their activity in presence of bitter compounds, and thus change the gustatory code^9,16–19^.

Subsequently if preventive taste-driven barriers are overridden and animals feed on noxious compounds, their ingestion can cause physiological constraints to animals. Ingestion of some alkaloids has shown lethal effects depending on the dose^20–23^. Some studies have shown also indirect effects of alkaloids ingestion in bees, reflected on a poor learning performance^21^, or changes in behavior characterized by more time spent standing still, grooming, abdomen dragging and curling up^24^. However, until now, there is no study where the direct effect of alkaloids on the metabolic rate of an insect was measured. Insects that undergo physiological constraints following the ingestion of toxic compounds might increase consequently the activity of enzymes associated with detoxification processes^25–27^. Thus, we hypothesize that these physiological processes could be reflected in an increase of the metabolic rate. Moreover, we predict that this increase would be reflected in a shift of the gas-exchange pattern from a discontinuous gas exchange cycle (DGC; generally associated with a low metabolic rate, *e.g*., at rest) to a continuous gas-exchange pattern (CGE; associated with high metabolic rate, *e.g*., by activity or stress). This sort of switch was observed in many insects that ingested insecticides, revealing the physiological stress suffered^28,29^. However, metabolic rate or gas-exchange pattern were never measured in an insect after ingesting alkaloids. Generally, “classical” DGC, in insects that mainly use their tracheal system for gas exchange, is composed of three phases or periods. A period called C (for closed), when spiracles are closed and no gas exchange between the atmosphere and the animal occurs. A second period called F (for flutter), when spiracles open and close for short periods of time. Finally a period called O (for open) when spiracles open to release accumulated CO_2_^30–33^. In contrast, the CGE the closing phase is absent and the spiracles remain open constantly, allowing CO_2_ to be released as it is produced therefore fulfilled higher O_2_ demands^34^

*Rhodnius prolixus* Stål 1859 (Hemiptera: Reduviidae) is a hematophagous insect that feeds mainly on vertebrate hosts, and is vector of Chagas disease. Since at present there are no vaccines against this disease, it is essential to focus efforts on the reduction of vector populations or on the improvement of personal protection tools^35^. Previous investigations showed that the addition of alkaloids to an appetitive solution negatively interferes with feeding^36^. However, the physiological mechanisms behind this aversive feeding response are not known. Thus, the aim of this work was to investigate whether alkaloids are merely distasteful, or they cause a physiological distress reflected as changes in metabolic parameters of insects, or both. To this end, we tested the feeding response of insects to quinine, caffeine, and theophylline at different concentrations. Caffeine and quinine detection produced both feeding inhibition in *R. prolixus*, as showed a previous study of our group^36,37^. However it is not known if they are just unpalatable compounds or have any impact in the physiology of this insect. On the other hand, the theophylline is an alkaloid with a similar structure to that of caffeine, but lacking of a methyl group. Following the ingestion of alkaloids, we measured the real-time CO_2_ production (as a proxy for metabolic rate) by open-flow respirometry, and registered the survival of insects.

## Results

### Feeding effect of alkaloid ingestion

**Figure 1:**
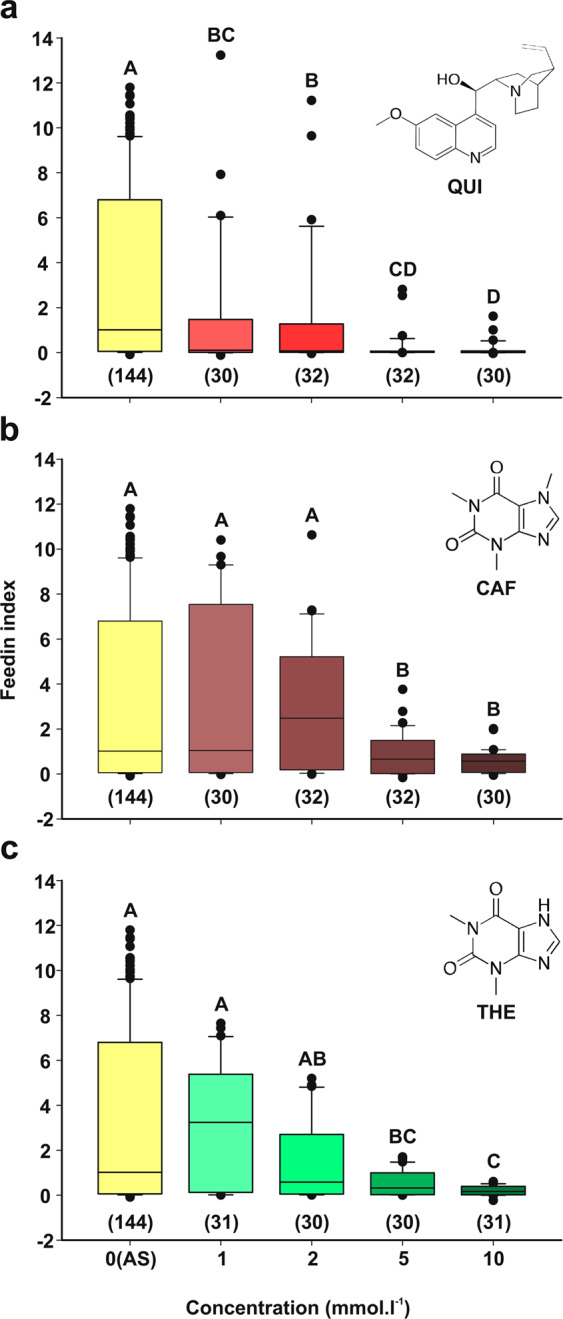
Aversive response to alkaloid ingestion in *R. prolixus*. The feeding performance of insects, measured as the feeding index (FI), depicted in function of the concentration of each alkaloid tested: (**a)** quinine (QUI), (**b)** caffeine (CAF), and (**c**) theophylline (THE). Next to each plot the molecular structure of each alkaloid is represented. All three alkaloids produced a dose-dependent anti-feedant response in insects. FI values were represented in box-plots, in which the median was represented as the separation line inside the box, which delimits from 25% to 75% of the data, the 95% and the 5% of data were depicted by the upper and lower whiskers and dots represent the outliers. The number of insects used for each solution was indicated between parentheses and different letters indicate statistical differences.

As previously shown^36^, *R. prolixus* fed on appetitive solution (AS) exhibiting median feeding index (FI) values of about 1, and some insects reached FI’s near 7 (Fig. 1). The FI shows the times an insect increases its initial body mass following the feeding assay. In contrast to the AS, the three alkaloids, *i.e*., quinine (QUI), caffeine (CAF) and theophylline (THE), produced aversive feeding behaviors that increased with the concentration of the compounds, sometimes reaching FI values near zero (Fig. 1a–c, QUI, H_(4)_ = 58.40, p < 0.0001, CAF, H_(4)_ = 13.31, p < 0.0099, and THE, H_(4)_ = 25.37, p < 0.0001). Based on this dose-dependence response, we calculated a threshold concentration, defined as the lowest concentration that significantly differed from AS. Threshold concentrations were 1 mmol.l^−1^ for QUI, and 5 mmol.l^−1^ for CAF and THE (*a posteriori* comparisons, AS *vs*. 1 mmol.l^−1^ QUI or 5 mmol.l^−1^ CAF or THE, p < 0.05).

### Metabolic impact of alkaloid feeding

Feeding solutions containing alkaloids were mostly avoided and generally insects probed the solutions several times, as they inserted and retracted their proboscis, sucking solution in each probe. In order to ensure that all insects had ingested similar amounts of solutions, we considered only those feeding events in which the insect’s proboscis remained inserted in the feeder during 2.5 minutes (see Materials and Methods) and that at the end of this time insects showed FIs around 1. Thus, the FI of QUI fed insects was 1.06 ± 0.06, 0.99 ± 0.04 for CAF, 0.90 ± 0.05 for THE, and 1.05 ± 0.06 for AS. Consequently, and because no statistical differences were found across treatments (F_(3, 36)_ = 1.846, p = 0.156) the metabolic measurements were carried out for each group.

**Figure 2:**
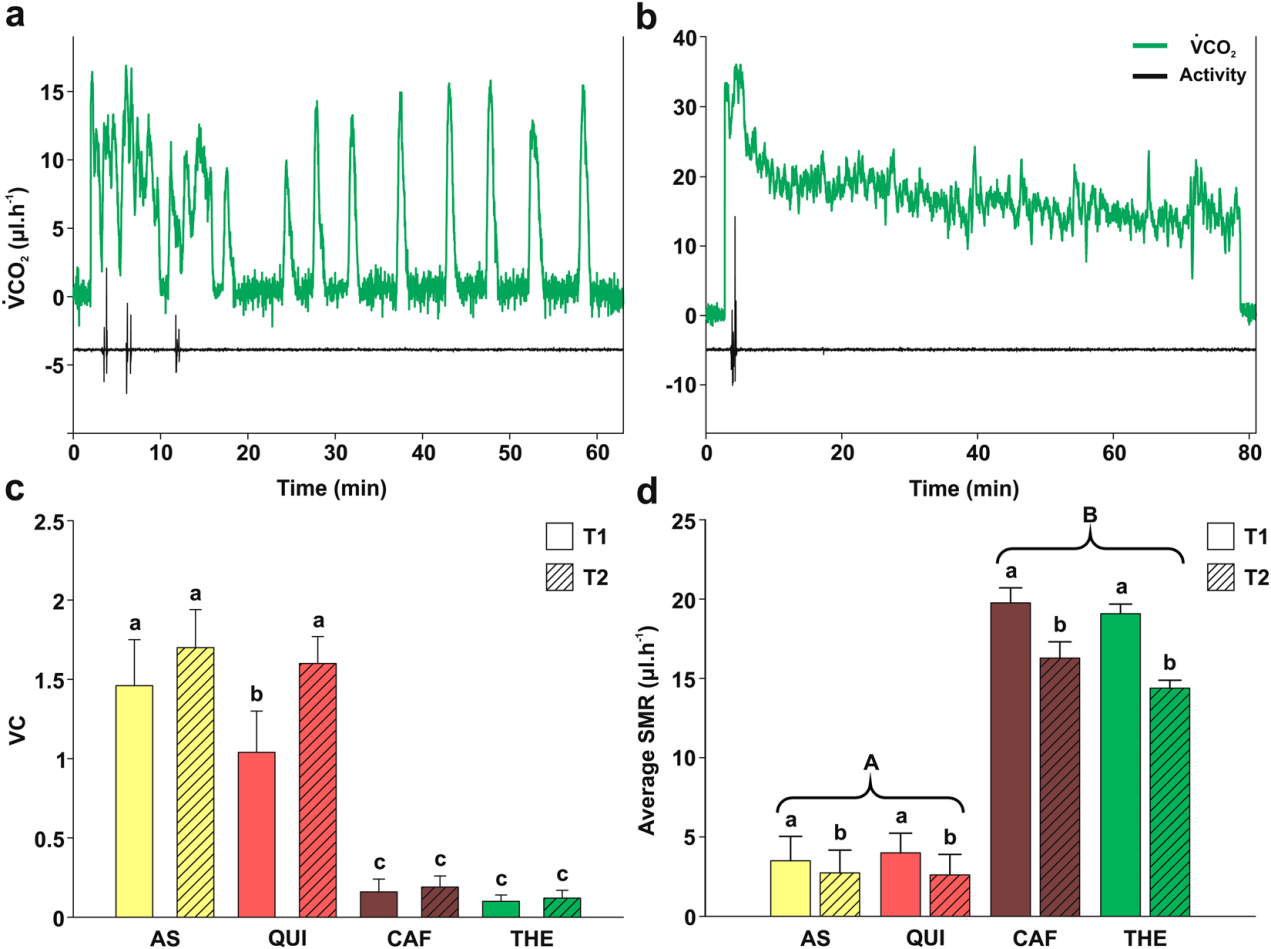
Metabolic impact after alkaloid ingestion in *R. prolixus*. (**a,b)** Examples of different CO_2_ emission patterns observed accordingly to the alkaloid ingested: (**a)** discontinuous gas exchange cycle (DGC) following QUI ingestion, (**b)** continuous gas exchange (CGE) observed after THE ingestion. Locomotor activity (black line) and CO_2_ emission rate (green line) traces are plotted as function of time (minutes). (**c)** Variation coefficient (VC) depicted in function of the ingested solutions and the post-ingestion time (T1: 5 min, T2: 3–5 h). AS and QUI showed higher VC values (even at different post-feeding times) than CAF and THE, these results quantitatively confirmed a DGC pattern for AS and QUI and a CGE pattern for CAF and THE. (**d)** Average standard metabolic rate (SMR) for the ingested solutions at two post-ingestion times as in (**c)**. Following CAF and THE ingestion insects significantly increased their SMR with respect to AS and QUI groups. VC and SMR are expressed as means ± SE. Ten individuals for each treatment were assayed. Lowercase letters represented post-ingestion time comparisons and uppercase letters indicated differences among experimental solutions.

The CO_2_ pattern release from different recordings across individuals within the same treatment was remarkably similar. All insects fed on AS and QUI solutions showed a discontinuous gas exchange patterns (DGC) (Fig. 2a), whereas all insects fed on CAF and THE exhibited a continuous gas exchange (CGE) pattern (Fig. 2b). In order to quantitatively differentiate between DGC (Fig. 2a) and CGE (Fig. 2b) patterns, the variation coefficient (VC) was calculated. Insects fed on the different solutions differed in their gas exchange VC (F_(7,72)_ = 161.66, p < 0.0001). Both CAF and THE showed significant lower VC for the two measured times, *i.e*., 5 minutes (T1) and 3–5 hours post-ingestion (T2), than AS and QUI solutions (Fig. 2c; *a posteriori comparisons*, p < 0.05). These results confirmed a CGE following ingestion of CAF and THE. The high VC values found for AS and QUI also confirmed a DGC gas exchange pattern (Fig. 2c; *a posteriori* comparisons, p < 0.05).

In Fig. 2d, the standard metabolic rate (SMR) of insects differed according to the solution ingested and the post-ingestion time. No interaction effects were detected (F_(3; 36)_ = 2.58, p = 0.06). It is important to note that, because there were no significant differences in the initial mass of insects across treatments (F_(3; 76)_= 2.03, p = 0.116, data not shown), there was no need to standardize the metabolic values for insect's mass. In addition, given that similar amounts of all solutions were ingested, the differences observed for the SMR can only be attributed to an effect caused by the experimental solutions, and not to the differences in the amount of food ingested or mass variations. Thus, the SMR varied depending on the experimental solution ingested (F(3; 36) = 70.65, p < 0.0001). Insects that ingested CAF or THE exhibited significantly higher SMR than those who ingested AS or QUI (Fig. 2d) (*a posteriori* comparisons, p < 0.05). Moreover, in all cases, the SMR was significantly lower at T2 than at T1 (F(1; 39) = 33.33, p < 0.0001; Fig. 2d).

### Effect of alkaloid ingestion on survival

**Figure 3:**
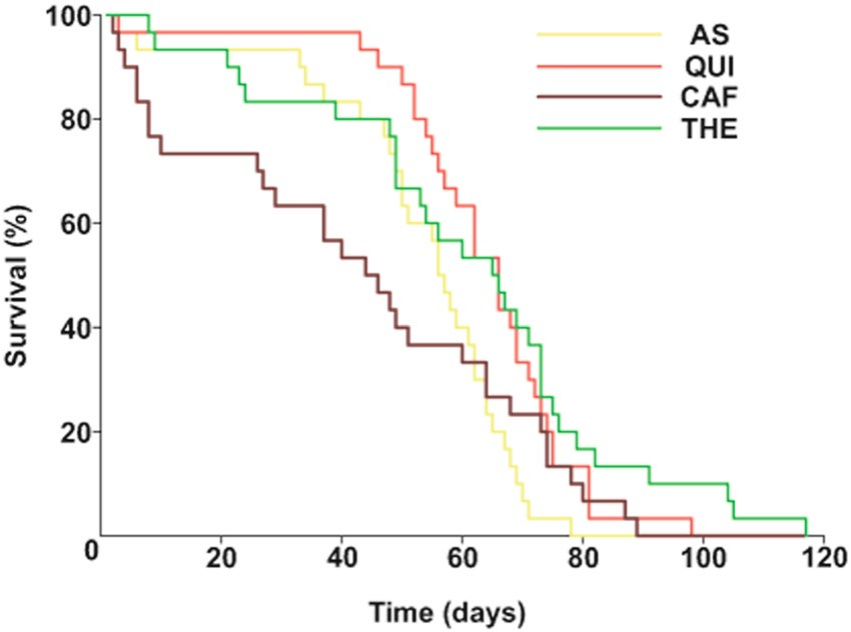
Kaplan-Meier curves of survival after alkaloid ingestion in *R. prolixus*. Percentage of live insects plotted as a function of time (days) after ingestion of AS or QUI, CAF and THE at threshold concentrations. Main differences across the survival curves of the four groups occurred during the first 40 days. CAF produced significantly higher mortality rate than the AS, QUI, and THE treatments. Thirty insects were used in all tests.

We also quantified insect survival following ingestion of AS and alkaloids along 116 days (Fig. 3). The comparison of the whole survival curves revealed significant differences across groups (long rank _(3)_ = 11.45, p = 0.01), with main differences occurring when comparing curves during the first 40 days (log rank _(3)_ = 18.64, p < 0.001). Therefore, insects that ingested CAF had a higher mortality rate (*a posteriori* comparisons, CAF *vs*. AS, Holm-Sidak = 6.31, p = 0.01, CAF *vs*. QUI, Holm-Sidak = 14.5, p = 0.0001, CAF *vs*. THE, Holm-Sidak = 4.94, p = 0.02). This remarkable effect was only evinced for CAF, which produced significantly more deaths than the AS, QUI, and THE. Also, during the first 40 days the survival curves of AS, QUI and THE groups were not statistically different from each other.

## Discussion

In this work, we established in *R. prolixus* the relationship between feeding on three different alkaloids and the physiological consequences. Quinine, caffeine, and theophylline produced anti-feedant responses, although depending on the alkaloid ingested, this avoidance behavior could be simply explained as a low palatability of chemicals, or as physiological contraints reflected in the metabolism of insects (*i.e*., gas exchange patterns and SMR), as well as in insect’s mortality.

### Effect of alkaloid on ingestion

Rising concentrations of any of the three alkaloids assayed increased feeding aversion (Fig. 1). However, quinine had an effect at a lower concentration than caffeine or theophylline. In addition to caffeine and quinine, feeding reluctance was already observed in *R. prolixus* when offering other bitter compounds such as salicine and berberine^36^. However, their physiological effect, if any, was unknown up to now. Aversive responses upon ingesting quinine or caffeine, have been reported for other hematophagous insects, such as mosquitoes^38–40^, and also for phytophagous insects such as flies and moths^10,11,41–46^.

Actually, it has been demonstrated that bitter detection modulates feeding decisions in many different mammals and insects^47,48^. Mostly, phytophagous insects have bitter-sensitive gustatory neurons tuned to detect plant toxins^17^ that often have a bitter taste for humans. The bitter sensitivity of an obligatory blood-sucking insect like *R. prolixus* is paradoxical. *R. prolixus* nymphs and adults feed almost exclusively on blood of vertebrates, a feeding source essentially lacking alkaloids unless ingested by their hosts^49^. In this case, alkaloids or bitter molecules can become an active part of their blood. Similar responses to quinine and caffeine are found in phytophagous insects of the same suborder (Heteroptera) in the genera *Dysdercus* and *Spilostethus*^50^. Moreover, *R. prolixus* evolved from predatory ancestors, for which the adaptive pressure to detect alkaloids was probably high. In fact, starved *R. prolixus* bugs can feed on hemolymph of arthropods^51^. Therefore, this might be a conserved trait^49^. Following external evaluation of food, by GRNs in the legs, proboscis and antennae, gustatory assessment of incoming food or blood occurs internally, through taste sensilla located inside the pharynx of insects^4,36,52–54^, etc. These internal sensilla are almost immediately bathed with food or blood as soon as the first gorge passes through the alimentary canal. Taste sensilla in the pharynx house GRNs tuned to phagostimulant and noxious or anti-feedant molecules. Thus, food quality is evaluated once inside the insect, before deciding whether to continue to feed or not. Certainly, the activation of one of these two opposite pathways drives appetitive or anti-appetitive responses, helping insects to feed on nutritious food and to avoid harmful or potential toxic food. However, the appetitive pathway can also be inhibited by the presence of potential noxious compounds. This inhibition mechanism might occur to prevent ingesting harmful substances occurring in mixtures^9,16–19^. Although less studied than external GRNs, the physiology of internal GRNs has become to be attended^4,9,52,54,55^. In contrast much less information is available about the taste sense of hematophagous insects. Based on morphological studies by means of scanning electron microscopy, eight taste sensilla were identified in the anterior part of the pharynx of *R. prolixus* ^36,49^. Likely, these sensilla are responsible for the gustatory evaluation of blood, following biting and sucking a gorge of blood in kissing bugs^36,56–58^. However, electrophysiological studies are necessary to corroborate their function as, for example, bitter-sensitive GRNs tuned to alkaloids. The anti-feedant effects of caffeine and theophylline were quite similar, although, both had different effects on insects’ survival (see below). The remarkably similar structure of these two compounds, which differ only in a methyl radical, make us wonder whether both molecules could be detected by the same gustatory receptor/s or not.

### Effect of alkaloid ingestion on metabolism

Our results showed that the ingestion of both, caffeine and theophylline, produced an increase (*ca*. 4–6 times) in their SMR compared to insects that ingested AS or quinine. Notably the SMR changed in all groups with post-ingestion time, being higher in insects recently fed. This increase in the SMR observed in all treatments, could be due to an undergoing diuresis process that normally begins soon after ingestion in *R. prolixus*^59,60^.

An enhancement of the SMR was also observed when treating different arthropods with insecticides. Such situation triggers a metabolic stress that increased the insect’s metabolic rate,^61–63^ likely due to a detoxification process. Metabolic rate depends on extrinsic factors such as temperature, and intrinsic factors such as the reproductive status, body mass, activity levels, and nutritional status of the animal^64,65^. In this study, all these factors were controlled: the temperature was constant, insects were sexually immature, their body mass was similar before and after treatments, CO_2_ production was quantified at rest (*i.e*., insects were immobile), and all insects had the same level of fasting. Therefore, all metabolic rate variations are necessarily caused by the ingestion of caffeine and theophylline. Detoxifying enzymes like esterases, glutathione S-transferases, and cytochromes P450 are involved in the detoxification processes^66^. Most of them become activated when animals, including insects, ingest plants that produce compounds with toxic effects. Thus, detoxifying enzymes provide animals with a defense mechanism and a protection system against intake of harmful food^66,67^. For example, it has been observed that ingestion of the alkaloid nicotine resulted in an increase in the activity of cytochrome P450 in *Manduca sexta* larvae^25,68,69^. Our results would suggest that a detoxification process induced by a stressful situation, such as the ingestion of caffeine and theophylline, reflected as an increase in the insect’s metabolic rate and on the gas exchange pattern. Our results also revealed differences in the gas exchange pattern of insects that ingested appetitive (*i.e*. AS) or anti-appetitive solutions. The metabolic rate after ingestion of alkaloids switched from a discontinuous DGC to a continuous CGE gas exchange pattern, revealed by the CO_2_ emission profiles and the low VC values registered for caffeine and theophylline in contrast to quinine or AS.

### Effect of alkaloid ingestion on survival

Insects had a half lifetime of 44-66 days regardless of the solution ingested. In insects fed on AS the mortality can be attributed to starvation and therefore, an increasing number of deaths along time was expected in this control group. Although theophylline produced an aversive feeding response and a higher SMR than the AS group, it did not affect the survival of insects with respect to AS. Caffeine, in contrast, caused the highest mortality observed, mainly at the beginning of the experiment. However, even if caffeine and theophylline appear to barely differ in their chemical structure, it is evident that these compounds produce different effects in survival. It is likely that insects that survive beyond 40 days achieved detoxification and probably ended up dying of starvation like in the AS group. Future experiments would be needed to study the SMR at longer times, *e.g*., 40 days, and the biochemical activity of the detoxifying enzymes.

Survival following the ingestion of alkaloids has been documented in other insects, where increasing doses of quinine, caffeine or theophylline in the food can reduce survival in comparison with a regular diet^20–23,70^. Besides, ingestion of bitter compounds can produce changes in animals’ behavior, causing seizures, exaggerated responses to stimuli, and tumbling, among others^24,71–73^. After ingestion of caffeine and theophylline, insects exhibited an altered locomotor behavior, most of the animals were knocked down keeping their legs retracted and showing a remarkable quiescence that could be interpreted as a symptom of intoxication. However, as days progressed, THE fed insects tended to re-gain their normal locomotion, while those who had ingested CAF did not recover.

## Conclusion

Our results showed that although quinine, caffeine, and theophylline triggered aversive feeding behaviors, both caffeine and theophylline had an impact on the physiology of insects, by modifying the insect’s metabolism and the gas exchange patterns, and with caffeine reducing the survival of the insects. Our results suggest that quinine triggers an aversive response, probably at the sensory level (pre-ingestive), whereas theophylline and caffeine also triggered a negative signal, but probably due to a toxic effect (post-ingestive). Based on our results, quinine could be considered exclusively as a low palatability compound, however, it cannot be discarded that caffeine and theophylline are also of low palatability for *R. prolixus*. The taste system not only promotes consumption of nutritive substances, but also prevents the consumption of harmful compounds, which could eventually lead to death. Consequently, animals can reject feeding on food sources with apparently no deleterious effect. However under certain circumstances, animals may accept noxious food if their energy budgets are low^74,75^, if there are not other feeding alternatives^76^, if after a long exposure with noxious compounds they undergo non-associative (habituation)^77^ or associative cognitive processes^78^. In addition, as postulated by Glendinning^1^, herbivores, that recurrently encounter bitter and potentially toxic compounds in their diet, have evolved a high tolerance to bitter compounds and dietary poisons. This might be an adaptation to avoid rejecting all foods that taste bitter and are not strictly or heavily toxic. This capacity could certainly allow them to access to empty niches^79^. On the other hand, carnivores or hematophagous animals (like *R. prolixus*) rarely encounter bitter and potentially toxic compounds in their diet therefore it is likely that they express a low tolerance to dietary poisons^1^. In this way, when insects decide whether to eat or not to eat, they certainly must prevent making wrong or lethal decisions. So, when food is not fully appetitive it would be better to avoid it.

## Materials and Methods

### Animals

Fifth-instar nymphs of *R. prolixus* were used in all experiments. Insects were obtained from a laboratory colony, reared at 28 ± 1 °C, ambient relative humidity, under a 12 h: 12 h light-dark cycle, and were handled according to the biosafety rules of the Hygiene and Safety Service of the University of Buenos Aires. Insects had no access to food after ecdysis to fifth instar. Experiments were carried out 15 ± 2 days post-ecdysis. Each nymph was used only once.

### Solutions

Feeding assays were carried out by using an appetitive solution (AS) of 1 mmol.l^−1^ ATP in 0.15 mol.l^−1^ NaCl. Caffeine anhydrous (CAF; Biopack, Buenos Aires, Argentina), theophylline (THE; Sigma-Aldrich, MO, USA), and quinine hydrochloride (QUI; Sigma-Aldrich, MO, USA) were added to the AS at different concentrations: 1, 2, 5, and 10 mmol.l^−1^. All solutions were prepared weekly and stored at −18 °C. In all cases, the pH of the solutions was verified and adjusted to 7 if necessary with 1 mol.l^−1^ NaOH.

### Artificial feeder

The feeding response of kissing bugs to different solutions was quantified by using an artificial feeder (further details in^36^). It consists of a feeding and an insect container. The feeding container is covered at its bottom end with a latex membrane and filled with the feeding solution. The insect is placed inside the insect container, which is covered with a tissue mesh. Insects can easily perforate both the membrane and the mesh to get access to the feeding solution. The feeding solutions were offered at 35 °C in order to motivate insects to feed. The experiment started when the two containers were placed in close contact, and lasted for 10 minutes. Each insect was weighted before and immediately after the end of each experiment, feces and urine were negligible. Insects’ weight gain was quantified as the difference between the mass after being exposed to the feeding solution (M_*f*_) and the mass before the treatment (M_*i*_). A feeding index (FI) was calculated (see Eq. 1).

1 $$({\rm{M}}f-{\rm{M}}i)/{\rm{M}}i$$

### Respirometer

An open-flow respirometer was used to evaluate the metabolic effect (further details in ^80^) of feeding on alkaloid compounds. For this purpose, insects CO_2_ real-time emission and locomotor activity were measured using a high-resolution respirometer (TR-2, Sable Systems International, Las Vegas, NV, USA) with a Li-Cor CO_2_ infrared analyzer (Li-Cor, LI-6251, Lincoln, NE, USA, resolution 0.1 ppm CO_2_). Briefly, CO_2_ and water-free air was pumped at 80 ml.min^−1^ through low-permeability tubing (Bev-A Line, to minimize errors associated with CO_2_ absorbance) reaching the respirometry chamber (volume ~13 ml), where a single insect was placed. Thereafter, the airflow passed through the respirometry chamber and loaded with the CO_2_ produced by the insect to finally reach the CO_2_ infrared analyzer. The temperature of the respirometry chamber was set and maintained at 28 °C with the aid of a peltier element cabinet (PTC-1, Sable Systems International, Las Vegas, NV, USA), a controller (Pelt-5, Sable Systems International, Las Vegas, NV, USA), and continuously measured by a thermocouple (TC-2000, Sable Systems International, Las Vegas, NV, USA, accuracy 0.2 and resolution 0.01 °C). Insect’s locomotor activity was measured by an activity detector (AD-2, Sable Systems International, Las Vegas, NV, USA). Analog outputs from analyzers were connected to an A/D converter (UI-2, Sable Systems International, Las Vegas, NV, USA, 16 bit basic accuracy 0.05%), stored on a PC and analyzed offline with ExpeData (Sable Systems International, Las Vegas, NV, USA).

### Experimental protocol

#### Series 1: Effect of alkaloid ingestion

Different concentrations of the alkaloids, CAF, THE, and QUI added to the AS or the AS alone (as control feeding solution), were offered to insects in the artificial feeder. The FI of insects was quantified as explained before.

#### Series 2: Metabolic impact of alkaloid ingestion

The lowest concentrations of CAF, THE, and QUI that produced a significant difference in feeding performance compared to AS, were chosen to evaluate the impact of alkaloids on the insects’ metabolism. Because feeding itself can modify the metabolic rate of insects^80,81^, it was first necessary to standardize the amount of solution ingested for each individual. Thus, an independent group of insects was allowed to feed on AS for different periods of time, *i.e*., 1, 2, 3, 5, and 10 minutes. The rationale of this experiment was to establish the average time needed for an insect to ingest one time its initial mass (Mi). Therefore, for each time a FI was calculated and then adjusted to a linear function (Fig. S1). The regression analysis revealed a time of 2.5 ± 1.9 min (mean ± SE) (F_(1; 48)_ = 81.05, p < 0.0001, R^2^ = 0.63). Consequently, for the following experiments, insects were allowed to feed on AS or on AS with threshold concentrations of CAF, THE and QUI for 2.5 min, in order to ensure that all insects ate similar volumes. This was corroborated by calculating the FI of each insect after feeding. Next, the metabolic rate of the 4 experimental groups was measured at two different post-ingestion times: 5 minutes (T1) and 3–5 hours post-ingestion (T2). Thus, we were able to evaluate the effects of these alkaloids a few minutes and about 4 hours after ingestion. Each metabolic recording began with 3–5 min before the insect was placed inside the chamber, thus, we were able to register the initial baseline of the recording. Then, we placed the insect inside the chamber, allowed the system to stabilize for 10 min and recorded the metabolic rate during 90 min. Following this time, the insect was removed from the chamber, and the final baseline recorded for 3–5 min.

#### Series 3: Survival assays

Insects used for metabolic measurements were maintained at rearing conditions (see below) and daily monitored to determinate their survival.

### Data analysis

All statistical analyses were performed with InfoStat v2011^82^ or R software^83^ with a significance level α < 0.05, except otherwise stated.

#### Series 1: Effect of alkaloid ingestion

Statistical differences between the FI calculated for each concentration and feeding solution were determined using the non-parametric Kruskal-Wallis test, followed by *a posteriori* Dunn’s multiple comparisons test when the global statistical analysis was significant.

#### Series 2: Metabolic impact of feeding on alkaloid solutions

Initial and final baselines were subtracted from each recording assuming a linear drift. Then, CO_2_ produced by the insect was registered as emission rates by transforming CO_2_ values from ppm to μl.h^−1^ (see the formula in^84^). For each insect the standard metabolic rate (SMR) was calculated from the CO_2_ emission rate of a region of the recording where the insect remained motionless and showed a stable pattern of CO_2_ release for at least 10 minutes. This is a minimal time to minimize error measurements due to the complex dynamics of gas mixture and the low flow rate used at the beginning of the experiments. In addition, we calculated a variation coefficient (VC, see Eq. 2) for each recording. In this way, the CO_2_ emission pattern, *i.e*., continuous or discontinuous, for AS and each experimental solution could be evaluated^80,85,86^. High values for VC indicate DGC, as the insect cyclically opens and closes its spiracles. Low VC values indicate a continuous CO_2_ release, as the spiracles remain constantly open.

2 $${\rm{VC}}=\frac{{\rm{s}}{\rm{.d}}.}{{\rm{Mean}}}$$

Data were analyzed by using a generalized lineal model (repeated measures ANOVA), thus, the SMRs obtained for each group and post-ingestion times (T1 and T2) were fitted with the “gls” function from the “nlme” package^87^. Feeding solutions and post-ingestional times were settled as categorical fixed variables. We used the lowest Akaike information criterion (AIC) model, which employs first order autoregressive variance and covariance matrix. In addition, we used the “VarIdent” function to model de variance for tests with each feeding solution. Since the analysis revealed no interactions, the variability provided by individuals was disregarded. A *posteriori comparisons* were carried out by Tukey test with the “multcomp” package^88^.

#### Series 3: Survival assays

Survival curves were built as Kaplan-Meier survival plots until all insects died^82^. Statistical analysis among feeding solutions were made with Log-rank test and pairwise multiple comparisons through the Holm-Sidak method.

## Supplementary information

Supplementary information.

## Data Availability

The data are available from the authors upon reasonable request.
