# Supplementary material for: Impact of alkaloids in food consumption, metabolism and survival in a blood-sucking insect
Source: Sci Rep. 2020 Jun 10;10:9443. doi: 10.1038/s41598-020-65932-y (PMC7287067; doi:10.1038/s41598-020-65932-y)
Supplement: Supplementary file 1 — Supplementary information. [file 41598_2020_65932_MOESM1_ESM.pdf]

Impact of alkaloids in food consumption, metabolism and survival in a blood-sucking insect

Ignacio J. Muñoz[1,2], Pablo E. Schilman[2*] and Romina B. Barrozo[1*]

[1] Grupo de Neuroetología de Insectos Vectores, Laboratorio Fisiología de Insectos, Instituto Biodiversidad Biología Experimental Aplicada, CONICET - UBA, Departamento Biodiversidad Biología Experimental, Facultad Ciencias Exactas y Naturales, Universidad de Buenos Aires, Buenos Aires, Argentina.

[2] Laboratorio de Ecofisiología de Insectos, Instituto de Biodiversidad y Biología Experimental y Aplicada, CONICET-UBA, Departamento de Biodiversidad y Biología Experimental, Facultad de Ciencias Exactas y Naturales, Universidad de Buenos Aires, Argentina.

*** Equal contribution and corresponding authors:**

Romina B. Barrozo (rbarrozo@bg.fcen.uba.ar)

Pablo E. Schilman (schilman@bg.fcen.uba.ar)

SUPPLEMENTARY FIGURE

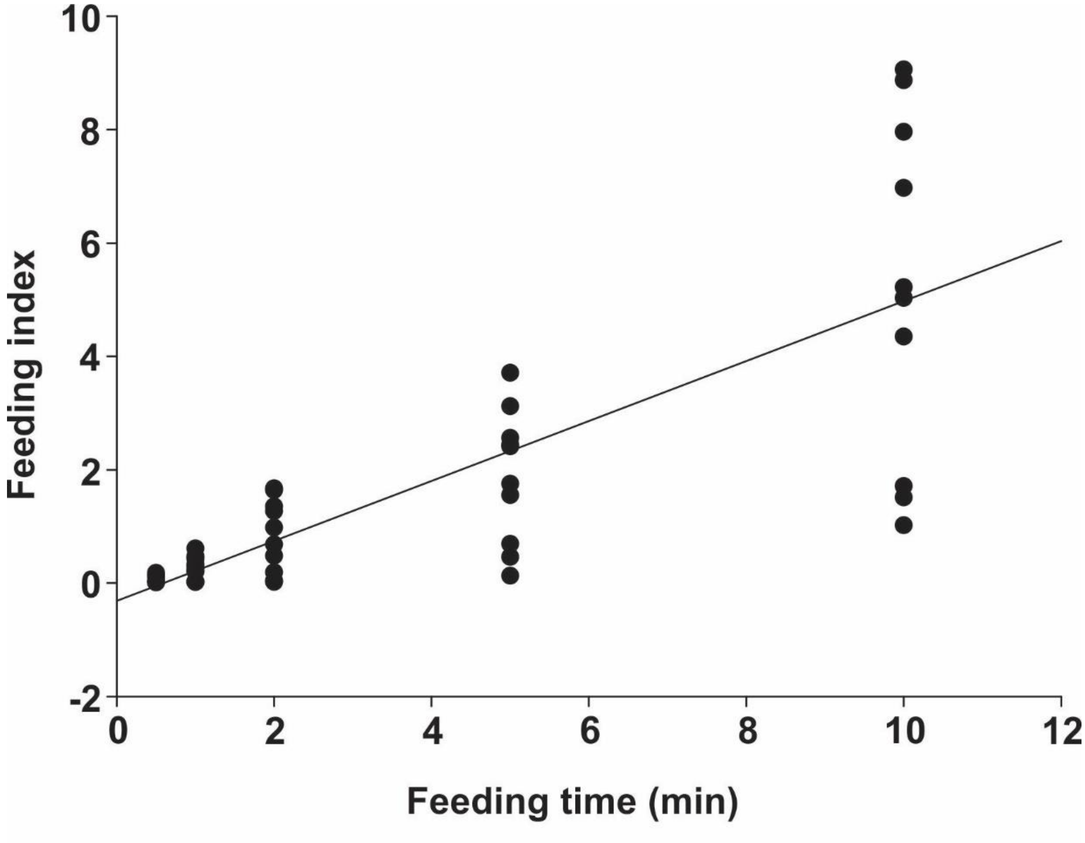

Figure S1. Standardization of needed time for *R. prolixus* to elicit a FI = 1. Feeding index (FI) displayed as a function of the net feeding time. The feeding time for a FI = 1 was 2.48 ± 1.96 minutes. To calculate this, a linear regression was computed with the "lm" function of the basic package of R. From this regression the time at which the FI is equal to 1 was obtained with the "inverse.predict" function of the "chemCal" package (Ranke et al., 2015). Ten insects were used for each time tested.

BIBLIOGRAPHY

Ranke, J., Ranke, M.J., Suggests, M., 2015. Package " chemCal ." Packag. " chemCal " 1–11.
